# Expression of Biphenyl Synthase Genes and Formation of Phytoalexin Compounds in Three Fire Blight-Infected *Pyrus communis* Cultivars

**DOI:** 10.1371/journal.pone.0158713

**Published:** 2016-07-13

**Authors:** Cornelia Chizzali, Asya K. Swiddan, Sahar Abdelaziz, Mariam Gaid, Klaus Richter, Thilo C. Fischer, Benye Liu, Ludger Beerhues

**Affiliations:** 1 Institute of Pharmaceutical Biology, Technische Universität Braunschweig, Mendelssohnstr. 1, 38106, Braunschweig, Germany; 2 Julius Kühn Institute, Federal Research Centre for Cultivated Plants, Institute for Resistance Research and Stress Tolerance, Erwin-Baur-Str. 27, 06484, Quedlinburg, Germany; 3 Biotechnology of Natural Products, Life Science Center Weihenstephan, Technische Universität München, Liesel-Beckmann-Str. 1, 85354, Freising, Germany; USDA/ARS, UNITED STATES

## Abstract

Pear (*Pyrus communis*) is an economically important fruit crop. Drops in yield and even losses of whole plantations are caused by diseases, most importantly fire blight which is triggered by the bacterial pathogen *Erwinia amylovora*. In response to the infection, biphenyls and dibenzofurans are formed as phytoalexins, biosynthesis of which is initiated by biphenyl synthase (BIS). Two PcBIS transcripts were cloned from fire blight-infected leaves and the encoded enzymes were characterized regarding substrate specificities and kinetic parameters. Expression of *PcBIS1* and *PcBIS2* was studied in three pear cultivars after inoculation with *E*. *amylovora*. Both *PcBIS1* and *PcBIS2* were expressed in ‘Harrow Sweet’, while only PcBIS2 transcripts were detected in ‘Alexander Lucas’ and ‘Conference’. Expression of the *PcBIS* genes was observed in both leaves and the transition zone of the stem; however, biphenyls and dibenzofurans were only detected in stems. The maximum phytoalexin level (~110 μg/g dry weight) was observed in the transition zone of ‘Harrow Sweet’, whereas the concentrations were ten times lower in ‘Conference’ and not even detectable in ‘Alexander Lucas’. In ‘Harrow Sweet’, the accumulation of the maximum phytoalexin level correlated with the halt of migration of the transition zone, whereby the residual part of the shoot survived. In contrast, the transition zones of ‘Alexander Lucas’ and ‘Conference’ advanced down to the rootstock, resulting in necrosis of the entire shoots.

## Introduction

Pear (*Pyrus communis*) has a cultivation history of more than 2000 years and is, after apple (*Malus domestica*), the second important fruit crop in the temperate regions of the world [1]. Production of pear fruits reaches approximately 25 million tonnes a year [2]. The great demand for the fruits is associated with their nutritional value and attractive taste [3]. Pears are high in vitamin C, potassium, iodine, and fibers. Pears are low in calories, stimulate digestion and bowel peristaltic movement, affect blood pressure, and exhibit diuretic, antipyretic, and antitussive activities [3, 4].

Common pear, also called European pear (*P*. *communis*), and Nashi pear (*P*. *pyrifolia)* are the two commercially important pear species. *P*. *communis* is popular in Europe, indicated by a number of cultivars. However, only few cultivars combine satisfactory appearance and flavor with long-term storability [3].

One of the major diseases of pear is fire blight, which is caused by the bacterial pathogen *Erwinia amylovora*. In Europe, fire blight is considered a growing problem because the increasing temperatures, the breeding of cultivars on susceptible rootstocks, and the introduction of susceptible cultivars into the market are likely to enlarge the risk of infection in the near future [5]. Due to the destructive character of the disease and the lack of effective control methods, sustaining a considerable fruit yield has become a major challenge in many parts of the world [6].

**Fig 1:**
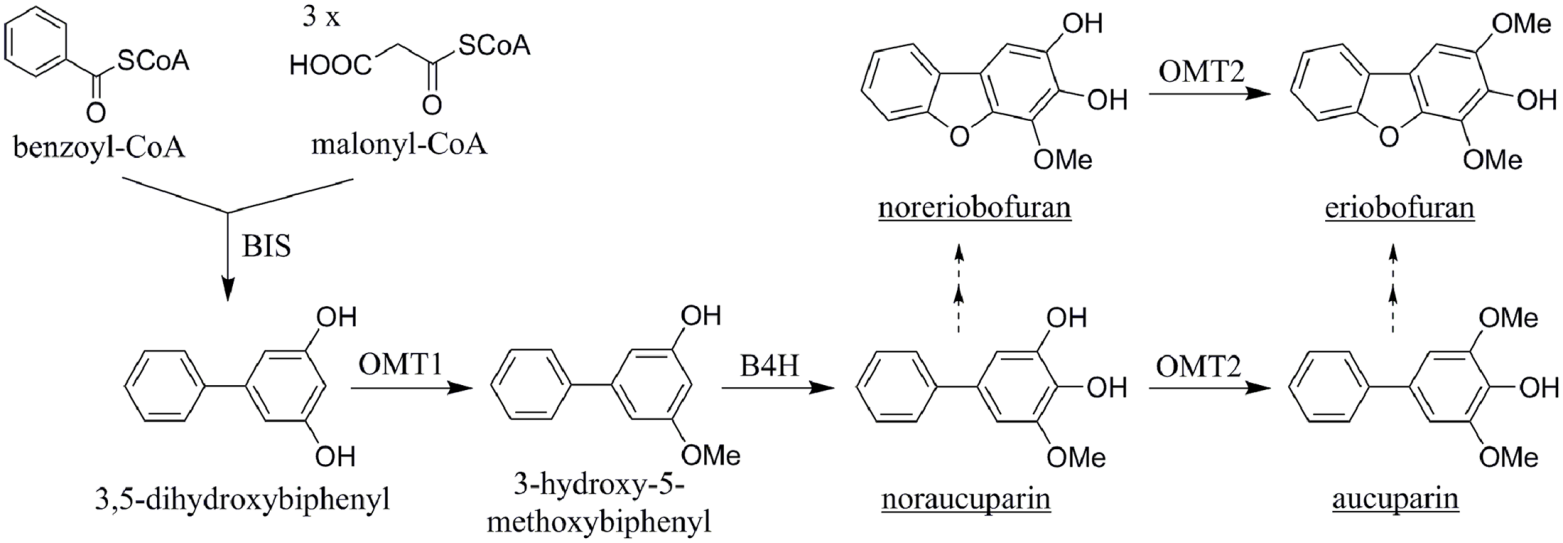
Biosynthesis of biphenyls and dibenzofurans. The biosynthetic pathway was proposed for phytoalexin formation in elicitor-treated *S*. *aucuparia* cell cultures [13]. Solid arrows, established reactions; broken arrows, postulated reactions. BIS, biphenyl synthase; OMT, *O*-methyltransferase; B4H, biphenyl 4-hydroxylase.

Formation of phytoalexins is one of the many complex and sequential responses of plants to pathogen infection [6]. Species of the Rosaceous subtribe Malinae, including pear, form biphenyls and dibenzofurans as inducible defense compounds [7]. Accumulation of these antimicrobial metabolites as *de-novo*-formed phytoalexins is confined to the Malinae. The biphenyl and dibenzofuran concentrations supposed to be present at localized infection sites inhibit spore germination and hyphal growth [8, 9]. The first enzyme of the biosynthetic pathway is biphenyl synthase (BIS), which was first detected in elicitor-treated *Sorbus aucuparia* cell cultures [10]. A BIS cDNA was then cloned and the recombinant enzyme was functionally expressed in *Escherichia coli* [11]. BIS catalysed the iterative condensation of benzoyl-CoA with three molecules of malonyl-CoA, yielding 3,5-dihydroxybiphenyl after decarboxylation (Fig 1). This intermediate undergoes conversion to aucuparin by two non-sequential *O*-methylation reactions, separated by a 4-hydroxylation step [12]. Recently, the two *O*-methyltransferase (OMT) enzymes have been studied in *S*. *aucuparia* cell cultures, including cDNA cloning, kinetic characterization, and homology modelling [13]. In addition, the cDNA for the cytochrome P450 enzyme, biphenyl 4-hydroxylase, has been isolated and functionally characterized [14]. Feeding of radioactively labelled 3,5-dihydroxybiphenyl to elicitor-treated *S*. *aucuparia* cell cultures demonstrated that dibenzofurans are derived from biphenyls, however, the underlying reactions are still hypothetical [13] (Fig 1).

Here, we report studies of phytoalexin biosynthesis in the three pear cultivars 'Alexander Lucas', 'Conference', and 'Harrow Sweet' after inoculation with *E*. *amylovora*. Two *PcBIS* genes were detected and their differential expression in response to the fire blight infection was investigated. In addition, formation of biphenyls and dibenzofurans was analyzed, allowing for correlation of the accumulation of the phytoalexins with the control of necrosis.

## Materials and Methods

### Plant material

Terminal shoots (45 cm) of the *Pyrus communis* L. cultivars 'Conference' and 'Harrow Sweet' were grafted on quince rootstock (*Cydonia* A) and grown in a greenhouse. A tree of the *P*. *communis* cultivar 'Alexander Lucas' (2 years old, 1.5 m high, grafted on quince rootstock) was purchased from a nursery in Blankenburg, Germany and also grown in a greenhouse. To achieve biological triplicates, shoots of all three tested cultivars were twice pruned back.

### Inoculation with *Erwinia amylovora*

Inoculation of the pear cultivars with the *E*. *amylovora* strain 222 gfp was carried out, as described previously [15].

### cDNA cloning of PcBISs

mRNA pools were isolated from fire blight-infected leaves and stems of *P*. *communis* 'Conference' and 'Harrow Sweet' using the RNeasy plant mini kit (Qiagen, Hilden, Germany) and reverse transcribed using RevertAid H Minus M-MuLV reverse transcriptase (Thermo Fisher Scientific, Waltham, MA, USA). Due to high identity between corresponding *P*. *communis*, *Malus domestica*, and *Sorbus aucuparia* sequences [11, 14, 16, 17] a degenerate primer pair covering the *S*. *aucuparia* and *M*. *domestica* BIS ORFs was designed (forward: 5-ATGGCGCCTTYGGTTAAGRATSA-3, reverse: 5-TTAGYATGKAATAGRTTCACTACG-3) and used to amplify PcBIS ORFs from the above cDNA pools. The PCR products were purified, cloned to the pGEM T easy vector (Promega, Madison, WI, USA), and sequenced (Eurofins Genomics, Ebersberg, Germany). The proofread sequence information is present in the GenBank/EMBL data libraries under the following accession numbers: *PcBIS1*, KU641483; *PcBIS2*, KU641484.

### Heterologous expression and enzyme purification and characterization

The PcBIS1 and PcBIS2 coding sequences were re-amplified by PCR using *Pfu* DNA polymerase (Thermo Fisher Scientific). The amplified sequences were cloned to the pRSET B vector (Invitrogen, Carlsbad, CA, USA) using the *Nhe* I and *Kpn* I sites. After sequencing the inserts on both strands, the recombinant plasmids were transferred for heterologous expression to *E*. *coli* BL21-CodonPlus (DE3)-RIL (Stratagene, Amsterdam, the Netherlands). The N-terminally His_6_-tagged proteins were purified to homogeneity using Ni-NTA agarose according to the manufacturer’s instructions (Qiagen). The purification efficiency was monitored by SDS-PAGE.

### PcBIS assay and kinetic studies

The standard assay (250 μL) contained either 6.8 μM benzoyl-CoA and 18.7 μM malonyl-CoA or 170 μM salicoyl-CoA and 841.5 μM malonyl-CoA in 0.1 M potassium phosphate (pH 7.0) and 2 μg protein. After incubation at 35°C for 20 min, the enzymatic products were acidified and extracted twice with ethyl acetate and analyzed, as described previously [11, 18]. The kinetic constants were determined using six substrate concentrations covering the range of 0.2 to 5x *K*_m_, while the concentration of the second substrate was kept constant at saturation. The incubation time was limited to 5 min. Hans-plot equation was used for calculating the kinetic parameters.

### *PcBIS* expression analysis by RT-PCR

Leaves and stems were collected at different times (0, 2, 5, 8, 12, 16, 21, 28, 35, and 42 d post-inoculation) and total RNA was isolated using the RNeasy plant mini kit (Qiagen). Reverse transcription of total RNA (1 μg) was carried out using RevertAid H Minus M-MuLV reverse transcriptase (Thermo Fisher Scientific) at 42°C. Gene-specific primer pairs were used to amplify core fragments of the PcBIS1 and PcBIS2 cDNAs (Table 1a). Actin (accession number AB190176.1) served as control for equal RNA amounts. PCR was carried out using *Taq* DNA polymerase (Peqlab, Erlangen, Germany). A denaturation step at 95°C (2:30 min) was followed by 30 cycles at 95°C (1:30 min), 58°C (30 s), and 72°C (1:30 min). The final extension was at 72°C for 10 min.

**Table 1:** Primers used for RT-PCR (a) and qRT-PCR (b).

a	Primer	Sequence 5'→3'
	Actin forward	ATG CCA TCC TTC GTC TGG ACC
	Actin reverse	AGC AGC TTC CAT TCC AAT GAG G
	BIS1 forward	GAG AGG CCG CTG TTT GAA ATT GTG
	BIS1 reverse	CTT TGC CTT CCC CAA TCG ATT TAT TTC
	BIS2 forward	GAG AGT CCA TTG TTT GAA ATC GTG GC
	BIS2 reverse	CTT TGC CTT CCT CAA TCG ACT TCT TTC
b	Primer	Sequence 5'→3'
	Actin forward	CTA TGT TCC CTG GTA TTG CAG ACC
	Actin reverse	GCC ACA ACC TTG ATT TTC ATG C
	BIS1 forward	TTG AAA TTG TGG CAT GCA GGC AGA CA
	BIS1 reverse	CAG GGT GCA CAC TTA AAA ATA AGG AA
	BIS2 forward	TTG AAA TCG TGG CAT GCA GAC AGA CG
	BIS2 reverse	CAG GGT GCA CAC TAA AAA ACA AGG AG

### *PcBIS* expression analysis by quantitative real-time PCR (RT-qPCR)

RT-qPCR was carried out on the Applied Biosystems 7500 Fast Real-Time PCR System (Applied Biosystems, Foster City, USA) using 5x HOT FIREPol EvaGreen qPCR Mix Plus (ROX; Solis BioDyne, Tartu, Estonia). cDNAs of two biological replicates were analyzed. All reactions were performed according to the procedure outlined in the manufacturer’s instructions, except that 45 cycles were used at 95°C for 30 sec and 63.5°C for 1 min. Gene-specific amplification was evaluated by melt curve analysis. Amplification and correlation efficiencies of each PCR reaction were determined using six serial dilutions of cDNA from inoculated pear stems ('Harrow Sweet', 8 dpi). The PCR efficiency was used to transform the Ct values into raw data for relative quantification. Expression of the *PcBIS1* and *PcBIS2* genes was evaluated using the primers listed in Table 1b. All samples were normalized using mRNA of the reference gene *Actin* (accession number DT002474) [19] as internal control sample for each line. The scaling of *PcBIS1* and *PcBIS2* expression was performed in relation to the respective mRNA levels in mock-inoculated leaves and stems (0 dpi), which were set to be one. All reactions were performed in technical triplicates. The efficiencies and the calculation of the expression levels were estimated using a published mathematical model [20].

### Extraction and analysis of biphenyl and dibenzofuran phytoalexins

Leaves and stems of each cultivar were collected at two different time points and freeze-dried. The collection times after inoculation with *E*. *amylovora* were days 12 and 21 for 'Alexander Lucas', days 16 and 28 for 'Conference', and days 12 and 28 for 'Harrow Sweet'. The extraction procedure, acidic and enzymatic hydrolyses, and GC-MS analyses were carried out, as described previously [21].

## Results

### cDNA cloning and functional analysis of PcBISs

Using a homology-based approach, two PcBIS transcripts were cloned from fire blight-infected leaves of the *P*. *communis* cultivar 'Conference'. The two coding sequences obtained comprised 1173 bp each and encoded 42.96 and 43.05 kDa proteins, which were named PcBIS1 and PcBIS2, respectively. The proteins shared 98.2% amino acid sequence identity with each other and 96.2% each with BIS1 from *S*. *aucuparia*. The coding sequences were functionally expressed in *E*. *coli*. The resulting N-terminally His_6_-tagged proteins were isolated by affinity-chromatography and their purity and subunit molecular masses were examined by SDS-PAGE (S1 Fig). When incubated with benzoyl-CoA and malonyl-CoA, both PcBISs formed 3,5-dihydroxybiphenyl as the major product and benzoyldiacetic acid lactone (6-phenyl-4-hydroxy-2-pyrone) as a minor by-product (S2 Fig). Identification of these products was carried out using HPLC-DAD and GC-MS (S3 Fig). The R_t_ values and spectra obtained matched those of the authentic reference compounds and agreed with literature data [11, 17]. Assays containing the heat-denatured proteins failed to produce the products. The pH optima of PcBIS1 and PcBIS2 were 7.5 and 7–7.5, respectively. The temperature optima were 30°C and 35°C, respectively. For both enzymes, increases in product formation were linear with the incubation time up to 30 min and the protein amount up to 4 μg in the standard assay. Beside benzoyl-CoA, both enzymes accepted salicoyl-CoA as starter substrate, leading to the formation of 4-hydroxycoumarin (S4 Fig). This enzymatic product was released after a single decarboxylative condensation, as observed previously with the *S*. *aucuparia* BISs [17]. Another starter substrate was 3-hydroxybenzoyl-CoA, yielding 3*-*hydroxybenzoyldiacetic acid lactone [6-(3'-hydroxyphenyl)-4-hydroxy-2-pyrone] as a single derailment product. 4-Hydroxybenzoyl-CoA was not accepted as starter substrate. Determination of the kinetic parameters (S5 and S6 Figs) demonstrated that PcBIS1 and PcBIS2 exhibited approximately 14- and 25-fold, respectively, higher turnover rates (*K*_cat_) with salicoyl-CoA than with benzoyl-CoA (Table 2). However, the *K*_m_ values for salicoyl-CoA were also 71- and 106-fold higher, respectively, than for benzoyl-CoA. As a result, the catalytic efficiencies of PcBIS1 and PcBIS2 with benzoyl-CoA were 5 and 4 times higher, respectively, than with salicoyl-CoA. Furthermore, the *K*_m_ values for malonyl-CoA strongly differed depending on whether either benzoyl-CoA or salicoyl-CoA were present.

**Table 2:** Steady-state kinetic parameters for the PcBISs with benzoyl-CoA and salicoyl-CoA as starter substrates.

Isoenzyme	Benzoyl-CoA			Malonyl-CoA^a^	Salicoyl-CoA			Malonyl-CoA^b^
	*K*_cat_	*K*_m_	*K*_cat_ */k*_m_	*K*_m_	*K*_cat_	*K*_m_	*K*_cat_*/k*_m_	*K*_m_
	[min^-1^]	[μM]	[M^-1^sec^-1^]	[μM]	[min^-1^]	[μM]	[M^-1^sec^-1^]	[μM]
PcBIS1	3.3±0.3	1.1±0.2	50000	13.7±2.9	46.2±4.5	78.4±4.8	9821	389.4±41.0
PcBIS2	2.1±0.1	0.9±0.1	38889	11.6±2.2	53.4±5.4	95.2±5.4	9349	326.7±16.8

^a^Determined in the presence of benzoyl-CoA,

^b^determined in the presence of salicoyl-CoA

### Phytopathological changes on pear shoots after *E*. *amylovora* inoculation

**Fig 2:**
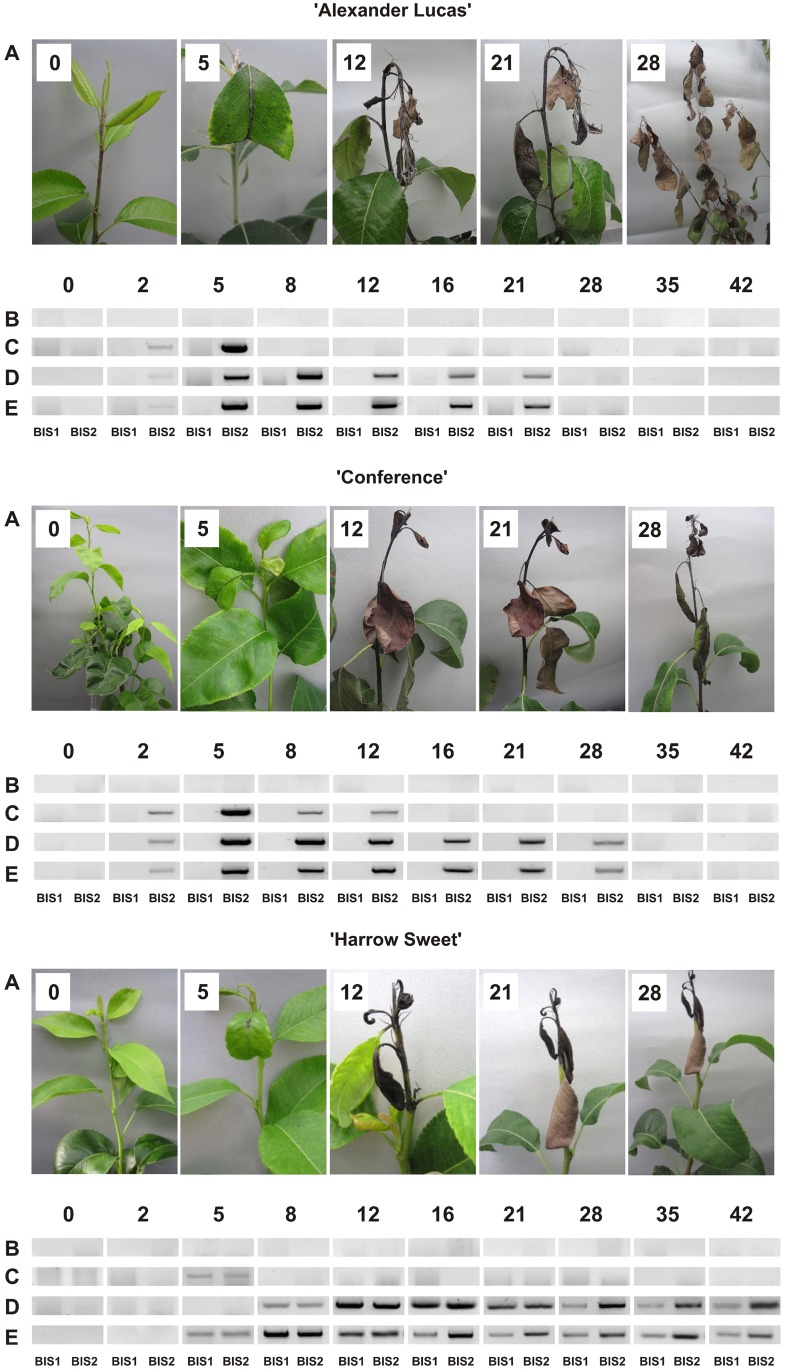
Phytopathological changes and *PcBIS* expression. Morphological changes (A) and expression of *PcBIS1* and *PcBIS2* (B-E) were studied in three pear cultivars after inoculation with *E*. *amylovora*. B: healthy leaf, C: leaf 1 (collectively necrotic leaves), D: leaf 2 (next leaf with a necrotic principal vein), E: transition zone (4-cm-stem segment between the necrotic and healthy parts).

Shoot tips of the cultivars 'Alexander Lucas', 'Conference', and 'Harrow Sweet' started to turn necrotic 2, 4, and 5 d post-inoculation, respectively, as indicated by necrosis of the principal veins in the top leaves, which were cut for inoculation (Fig 2). The necrosis then embraced the stem tips, and a transition zone between this necrotic and the healthy stem segments developed. The transition zone gradually advanced downward the stem, paralleled by necrosis of the leaves. Twelve days after *E*. *amylovora* inoculation, the top 15, 12, and 4 cm of shoots of 'Alexander Lucas', 'Conference', and 'Harrow Sweet', respectively, were necrotic (Fig 2). At this time point (12 dpi), the migration of the transition zone in shoots of 'Harrow Sweet' stopped. In contrast, shoots of 'Alexander Lucas' and 'Conference' suffered complete necrosis after 28 and 35 d of infection, respectively.

### Expression of *PcBIS1* and *PcBIS2* in *E*. *amylovora*-inoculated pear shoots

Fire blight-infected shoots of 'Alexander Lucas', 'Conference', and 'Harrow Sweet' as well as the corresponding mock-inoculated control shoots were collected at ten time points following inoculation and dissected from the tip downwards, as follows. Necrotic leaves were collectively referred to as leaf 1, the following leaf having a necrotic principal vein was designated as leaf 2. Another sample was the first healthy leaf. In addition, the transition zone of the stem, i.e. a 4-cm segment, was harvested. Up to day 8, all the three pear cultivars had no completely necrotic leaf. Therefore, the leaves that were cut for inoculation and had necrotic veins were analyzed as leaf 1 and the next yet healthy leaf was examined as leaf 2. The mRNA pools were isolated and used for reverse transcription (RT)-PCR. Furthermore, selected samples were employed for quantitative real-time PCR (RT-qPCR). Gene-specific primer pairs were designed and led to amplification of 385 bp (RT-PCR, Table 1a) and 210 bp (RT-qPCR, Table 1b) fragments of the PcBIS1 and PcBIS2 transcripts.

**Fig 3:**
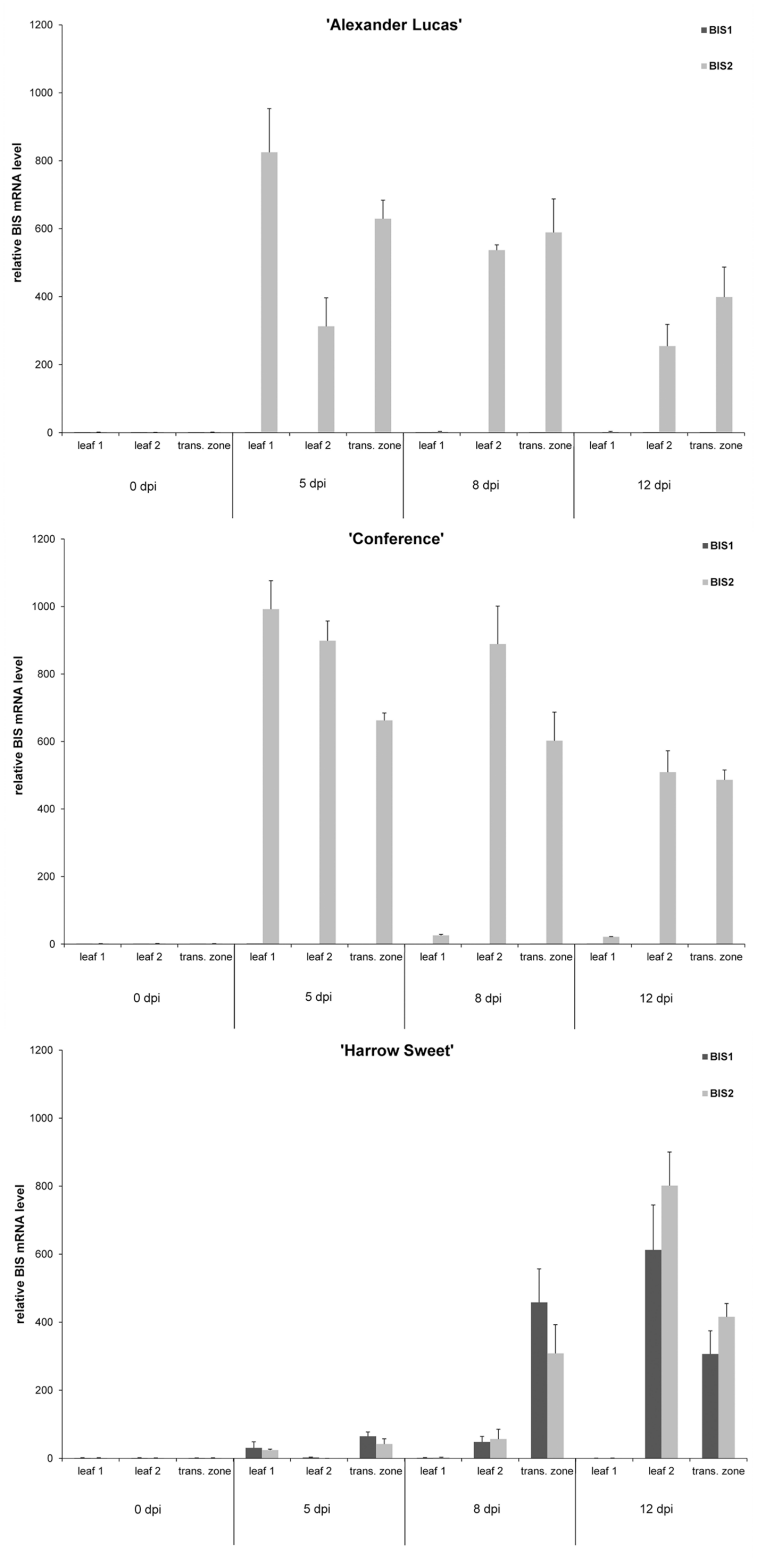
Transcript levels of PcBISs. Using RT-qPCR, relative transcript levels of PcBIS1 and PcBIS2 were determined in leaf samples and transition zones of three pear cultivars at day 5, 8, and 12 after inoculation with *E*. *amylovora*. Control mRNA was obtained from leaves and stems of mock-inoculated shoots (0 dpi). Leaf 1, collectively necrotic leaves; leaf 2, next leaf with a necrotic principal vein; transition zone, 4-cm-stem segment between the necrotic and healthy parts.

In 'Alexander Lucas', no BIS1 transcripts were detected using RT-PCR and RT-qPCR. In contrast, *BIS2* expression was observed 2 d post-inoculation and transcripts were detected in leaf 1, leaf 2, and the transition zone (Fig 2B–2D, 'Alexander Lucas'). Highest BIS2 transcript levels were found at day 5 in the leaves with a necrotic vein (leaf 1) as well as in the transition zone. *BIS2* expression in leaf 1 was 825 times that in the mock-inoculated control leaf (0 dpi), as demonstrated by RT-qPCR (Fig 3). At day 8, the first two leaves were necrotic and transcripts were no longer detectable, however, the BIS2 transcript level peaked in the leaf having the necrotic vein (leaf 2). Thereafter, *BIS2* expression decreased in both leaf 2 and the transition zone up to day 21. The entire pear shoot subsequently turned necrotic and expression of *PcBIS* genes was no longer detectable.

In 'Conference', the *BIS2* expression patterns for leaf 1, leaf 2 and the transition zone resembled those of 'Alexander Lucas', however, necrosis of the entire shoot took one week longer, and low BIS2 transcript amounts were still detectable at day 28 in leaf 2 and the transition zone (Fig 2B–2D, 'Conference'). The *BIS2* expression levels in 'Conference' were slightly higher than those in 'Alexander Lucas'. The maximum transcript level, found in leaf 2 at day 5, was 990 times that in the control leaf (Fig 3). Comparable to 'Alexander Lucas', no BIS1 transcripts were detected.

In contrast to the above two cultivars, 'Harrow Sweet' displayed both *BIS1* and *BIS2* expression, which started at day 5 in leaf 1 and in the transition zone (Fig 2B–2D, 'Harrow Sweet'). The highest expression levels were detected for *BIS1* at day 8 in the transition zone (Fig 3; approx. 460-fold up-regulation relative to the control) and for *BIS2* at day 12 in leaf 2 (approx. 800-fold up-regulation). Transcripts for both PcBISs were observed up to day 42 (Fig 2, 'Harrow Sweet'). The migration of the transition zone stopped after 12 d when the youngest four leaves were necrotic. After 21 d, the fifth leaf had a necrotic vein but afterwards the morphological appearance did not change until day 42.

In all three pear cultivars studied, no expression of *PcBIS* genes was detected in the healthy leaves.

### Biphenyl and dibenzofuran formation in *E*. *amylovora*-inoculated pear shoots

The transition zones (4 cm) of the three cultivars were collected for phytoalexin analysis. In addition, three leaves located at this transition zone (referred to as leaves 1, 2, and 3 from the tip) were analyzed. Corresponding mock-inoculated plant materials served as controls. Samples of 'Alexander Lucas' were collected at day 12, when the maximum *BIS2* expression was over, and at day 21, i.e. one week before the necrosis embraced the whole shoot. Neither leaves nor transition zones of infected plants contained detectable quantities of biphenyl and dibenzofuran phytoalexins. This was also true for the samples from mock-inoculated plants.

**Fig 4:**
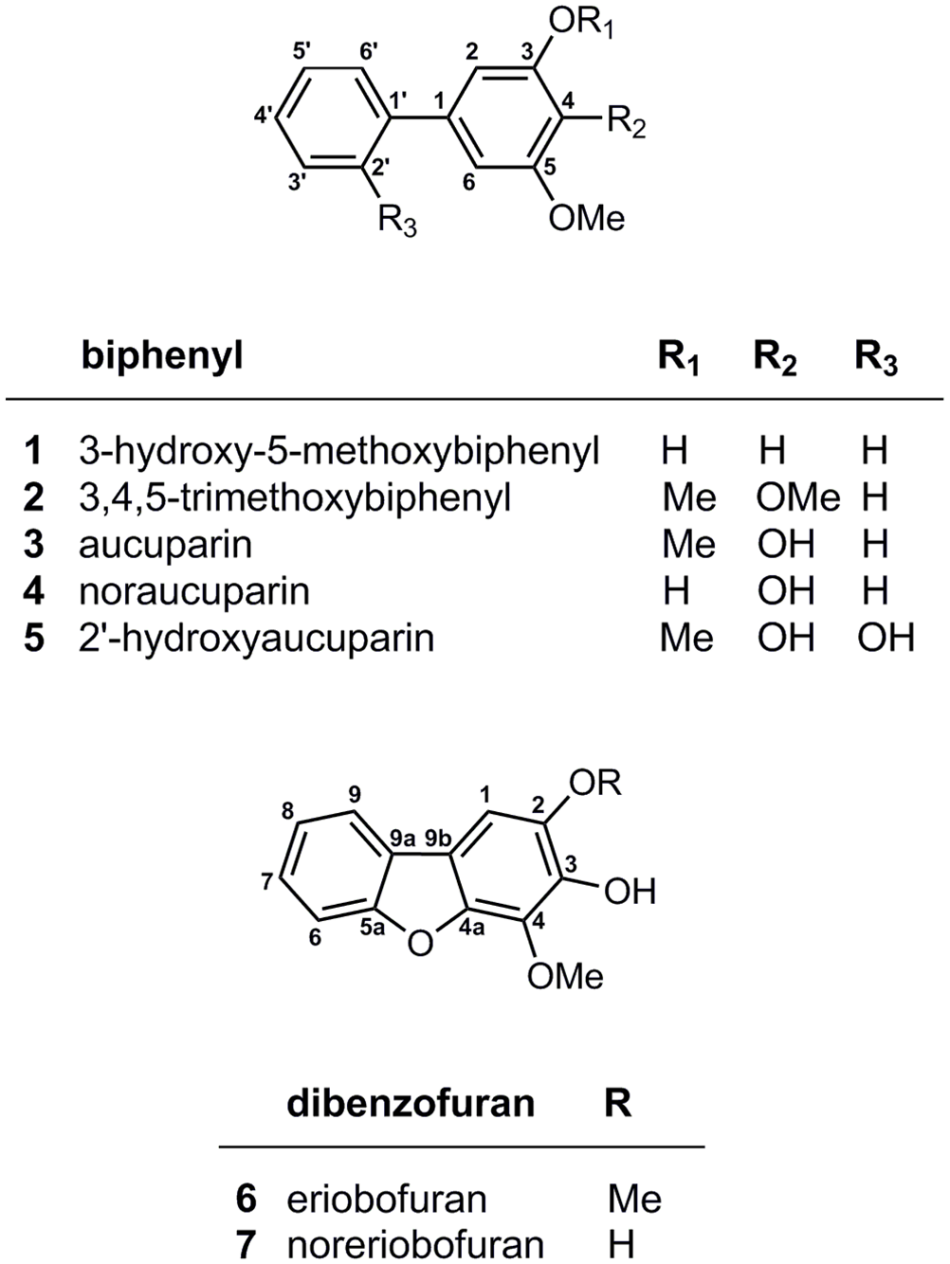
Chemical structures of biphenyls and dibenzofurans. The compounds depicted were detected in *E*. *amylovora*-inoculated shoots of 'Conference' and 'Harrow Sweet'.

**Fig 5:**
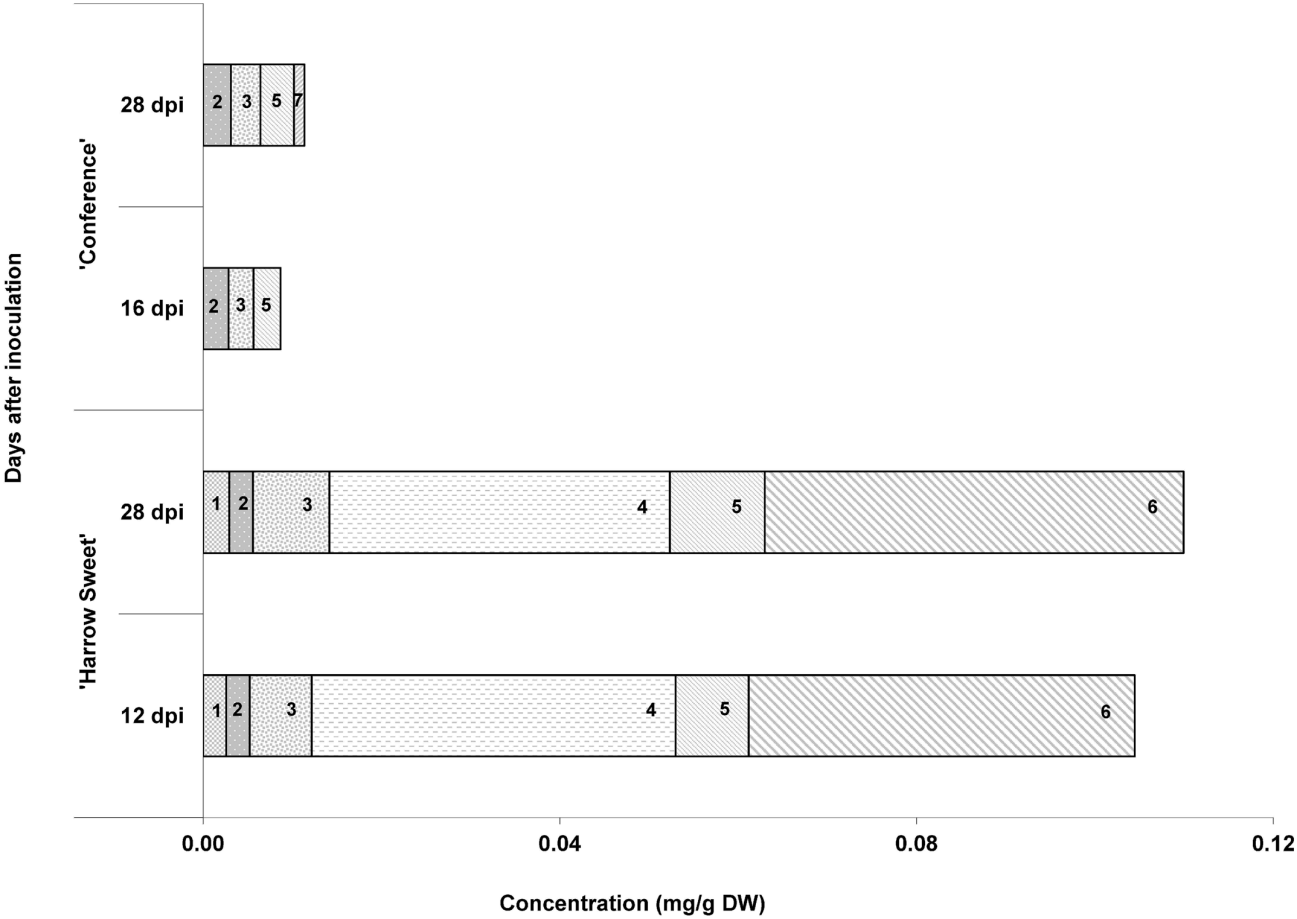
Biphenyl and dibenzofuran levels in transition zones. Concentrations were determined in the transition zones of fire blight-infected stems of 'Conference' and 'Harrow Sweet' at two time points post-inoculation (dpi). Data are average values ± SD of three independent experiments. Compounds are numbered according to Fig 4.

Samples of 'Conference' were harvested after 16 and 28 d. In the 28-day-old transition zone, three biphenyls were detected: 3,4,5-trimethoxybiphenyl (**2**), aucuparin (**3**), and 2'-hydroxyaucuparin (**5**; Fig 4). The dibenzofuran noreriobofuran (**7**) was also observed. Mock-inoculated plants lacked these compounds. The total phytoalexin content was 11.35 ± 6.12 μg/g dry weight (DW). At day 16, the dibenzofuran (**7**) was not yet detectable and the concentration of the three biphenyls was 8.69 ± 3.43 μg/g DW (Fig 5). All leaf samples analyzed were devoid of detectable phytoalexin amounts.

**Fig 6:**
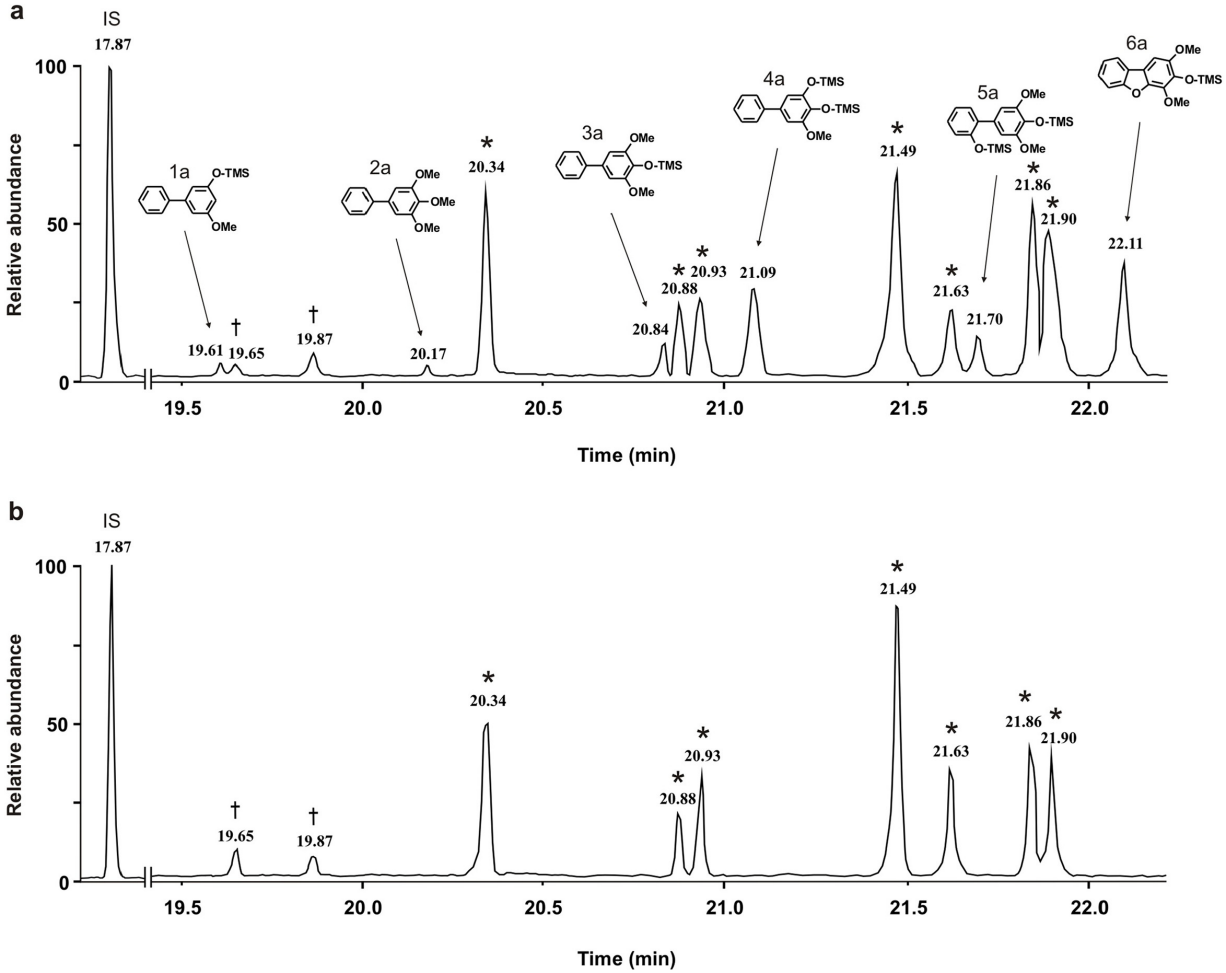
GC-MS analysis of biphenyls and dibenzofurans. The methanolic extracts studied were prepared from shoots of 'Harrow Sweet' 28 d after either infection with *E*. *amylovora* (a) or mock-inoculation (b). Compounds were separated as trimethylsilyl (TMS) derivatives named 1a–6a. IS, internal standard (4-phenylphenol), ^†^sugar derivatives, *fatty acid derivatives.

**Fig 7:**
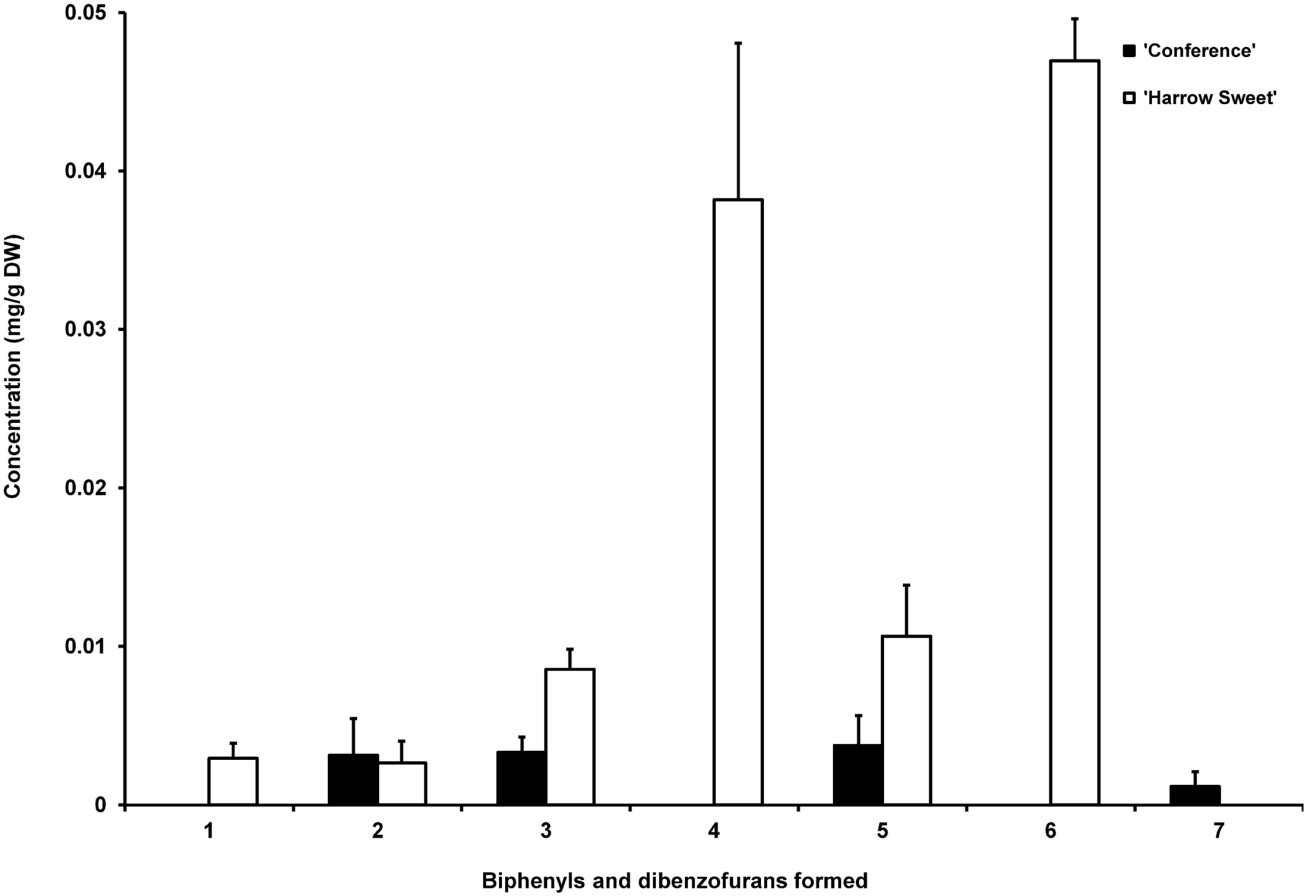
Array of phytoalexins in 'Conference' and 'Harrow Sweet'. Methanolic extracts stem from shoots collected 28 d after inoculation with *E*. *amylovora*.

Transition zones and leaves of 'Harrow Sweet' were harvested at day 12, when the migration of the transition zone stopped, and at day 28, when expression of the *PcBISs* was still relatively high. The biphenyls **2**, **3**, and **5** and in addition 3-hydroxy-5-methoxybiphenyl (**1**) and noraucuparin (**4**) were detected in the transition zone (Fig 6). Dibenzofuran **7** was replaced with eriobofuran (**6**). The total phytoalexin contents in 12- and 28-day-old transition zones were 104.46 ± 48.42 and 109.93 ± 19.36 μg/g DW, respectively (Fig 5). The detection limits for **3**, **4**, and **6** were 1.9, 1.6, and 7.6 μg/g DW, respectively, as determined previously [22]. The phytoalexins were absent from mock-inoculated plants. While 'Conference' formed the biphenyls **2**, **3**, and **5** in comparable amounts and the dibenzofuran **7** as a minor compound, 'Harrow Sweet' accumulated high concentrations of biphenyl **4** and dibenzofuran **6**, which were absent from 'Conference' (Fig 7). In the necrotizing leaves analyzed, no biphenyls and dibenzofurans were detected.

## Discussion

In pear, biosynthesis of biphenyls and dibenzofurans is induced by fire blight infection. The carbon scaffold of these phytoalexins is formed by BIS, which was found to be encoded by two differentially regulated genes, named *PcBIS1* and *PcBIS2*. While expression of *PcBIS1* was only detected in the cultivar 'Harrow Sweet', transcripts for PcBIS2 were observed in all three cultivars studied. In 'Harrow Sweet', the expression profiles of *PcBIS1* and *PcBIS2* were similar. The relative transcript levels increased up to 800-fold after *E*. *amylovora* inoculation, in the other two cultivars even up to 1000-fold, as determined by RT-qPCR. Previously, differential regulation of *BIS* genes was observed in the apple cultivar 'Holsteiner Cox', whose *BIS* gene family consists of four subfamilies, *MdBIS1* to *MdBIS4* [15]. The fire blight-induced accumulation of biphenyl and dibenzofuran phytoalexins was attributed to expression of *MdBIS3*. Transcripts for MdBIS3 were only detectable in the transition zone of the stem. The same was true for the phytoalexins. *MdBIS2* was expressed in leaves of fire blight-infected shoots; however, the transcripts were not translated into the protein, as demonstrated by SDS-PAGE and subsequent immunoblotting. In consequence, leaves were devoid of phytoalexins. Similar observations which suggest both transcriptional and translational regulation were made with the *BIS* genes of pear. In 'Harrow Sweet' with a high phytoalexin content, fire blight infection induced expression of *PcBIS1* and *PcBIS2* in both the transition zone of the stem and the necrotizing leaves, albeit the upregulation was faster in the transition zone. However, biphenyl and dibenzofuran phytoalexins were only detected in the transition zone. The same was true for 'Conference', where the timing of the PcBIS2 transcript upregulation in transition zone and leaves was similar. Unlike biphenyl and dibenzofuran metabolism, flavonoid formation in leaves of 'Conference' was affected by fire blight infection [6]. An increase in epicatechin after 3 d was associated with a preceding increase in the transcript levels of flavonoid biosynthetic genes.

The concentrations of the phytoalexins differed greatly in the three cultivars studied. In 'Harrow Sweet', the total phytoalexin level was almost ten times that in 'Conference'. The cultivar 'Alexander Lucas' even lacked detectable amounts of biphenyls and dibenzofurans. Notably, the phytoalexin level correlated with the degree of necrosis. In infected shoots of 'Alexander Lucas' and 'Conference', the transition zone advanced downward the entire stem up to the rootstock and hence the complete shoot turned necrotic. In contrast, the transition zone in diseased stems of 'Harrow Sweet' stopped its migration after 12 d when only the top 4 cm of the shoots were necrotic. The residual part of the shoots survived. The phytoalexin level in 'Harrow Sweet' was approximately a fourth of that in apple 'Holsteiner Cox' (430 μg/g DW) [21]. In infected stems of this apple cultivar, the migration of the transition zone also stopped but only after 42 d. Consistently, the same timespan passed to reach the maximum phytoalexin level in 'Holsteiner Cox'. In contrast, 'Harrow Sweet' needed only 12 d to accumulate high phytoalexin concentrations, which correlated with high PcBIS1 and PcBIS2 transcript levels between 8 and 12 d. Thus, reaching the maximum phytoalexin level may prompt the transition zone to stop the migration. Whether or not there is a threshold concentration remains open.

The major phytoalexins formed in 'Harrow Sweet' were the biphenyl noraucuparin and the dibenzofuran eriobofuran. Recently, noraucuparin was proposed to represent a metabolic branch point, from which formation of eriobofuran via noreriobofuran originates [13] (Fig 1). Both noraucuparin and noreriobofuran were accepted as substrates for SaOMT2, yielding aucuparin and eriobofuran, respectively. Noraucuparin results from hydroxylation of 3-hydroxy-5-methoxybiphenyl, catalyzed by the cytochrome P450 enzyme biphenyl 4-hydroxylase, which was heterologously produced in yeast and tobacco [14]. The hydroxylase exhibited absolute specificity for 3-hydroxy-5-methoxybiphenyl. The dibenzofuran-forming enzymes which convert either noraucuparin to noreriobofuran or aucuparin to eriobofuran have not yet been detected. The reactions likely involved are 2'-hydroxylation and subsequent cyclization [13, 23, 24]. Incorporation of the *ortho*-hydroxy group at the level of the BIS step was ruled out because the salicoyl-primed reaction undergoes only a single decarboxylative condensation with malonyl-CoA to yield 4-hydroxycoumarin rather than 2',3,5-trihydroxybiphenyl [15, 17]. The cyclization reaction on the 2'-hydroxylated intermediate may proceed via oxidative phenol coupling, as previously detected in xanthone metabolism [25]. Very recently, two P450 cDNAs for bifunctional enzymes were isolated, which catalyze both the hydroxylation and the subsequent regioselective cyclization reactions in xanthone biosynthesis [26].

**Fig 8:**
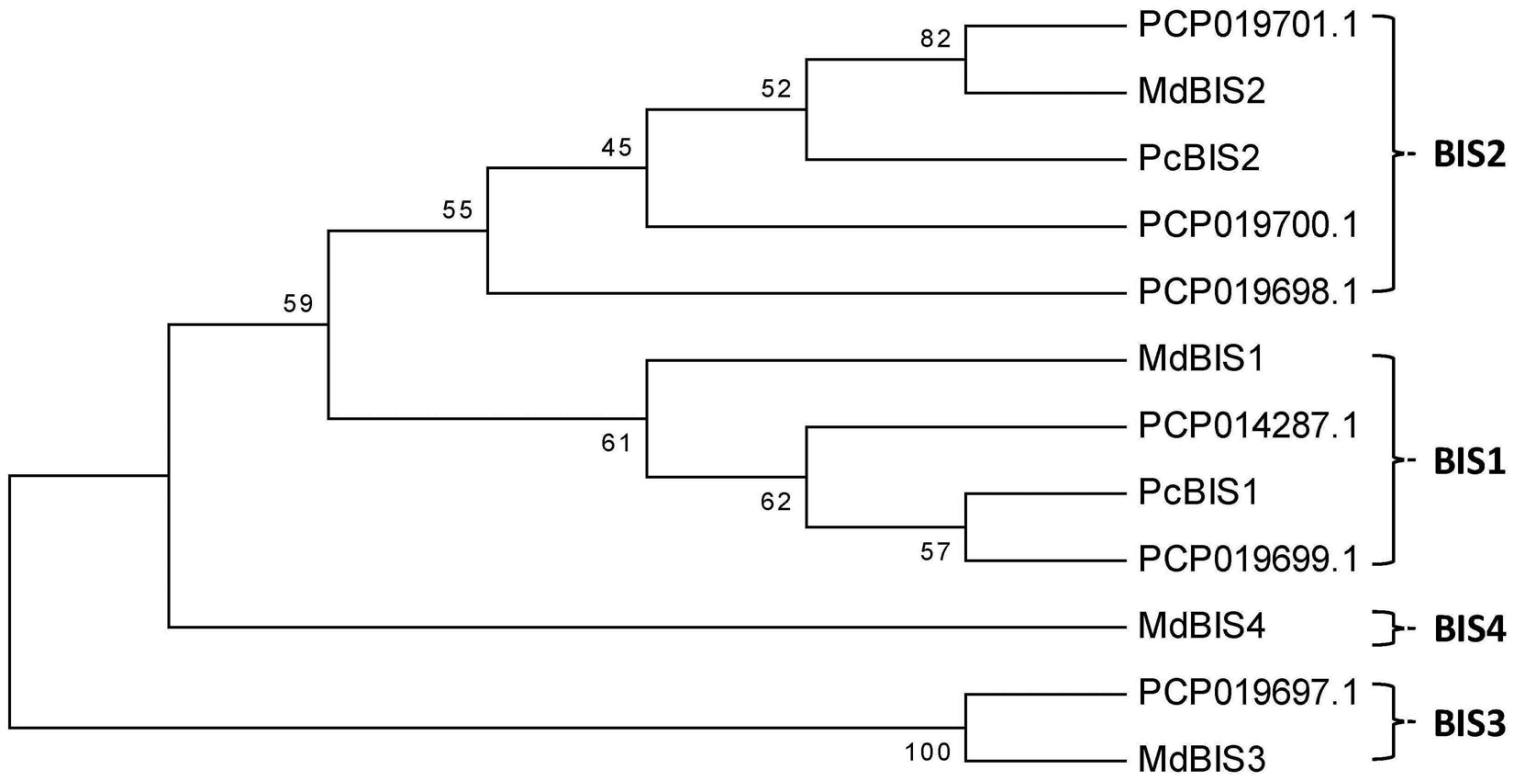
Neighbour-joining tree of PcBISs and MdBISs. The amino acid sequences were used for tree construction by MEGA 6.0. Numbers at the forks are percentage values of the bootstrap test (500 replicates). The accession numbers of the genes are: *MdBIS1*, JQ390521; *MdBIS2*, JQ390522; *MdBIS3*, JQ390523; *MdBIS4*, JQ390524.

Recently, the genome sequence of pear has been published (Rosaceae Genome Database) [27]. A genome-wide search for *BIS* sequences revealed that *PcBIS1* and *PcBIS2* are members of a gene family (Fig 8). In a phylogenetic tree, which also included the MdBIS amino acid sequences from apple, PcBIS1 grouped together with two PcBISs and MdBIS1. PcBIS2 clustered together with three PcBISs and MdBIS2. While a counterpart for *MdBIS4* was not found in the pear genome sequence, MdBIS3 grouped together with a yet unknown PcBIS3 sequence. In apple, *MdBIS3* is the only gene that is expressed in the transition zone and hence involved in the biosynthesis of biphenyl and dibenzofuran phytoalexins. In pear, this function appears to be taken over by the *PcBIS1* and *PcBIS2* subfamilies in 'Harrow Sweet' and the *PcBIS2* subfamily in 'Conference'. Future experiments will seek to reveal the conditions, under which *PcBIS3* may be expressed.

## Supporting Information

S1 Fig Overexpression and purification of PcBISs. PcBIS1 and PcBIS2 were overexpressed in *E*. *coli* and purified by affinity chromatography on Ni-NTA agarose. 1, pre-induction; 2, post-induction; 3, soluble protein; 4, affinity-purified protein; M, protein marker.
(TIFF)

S2 Fig HPLC chromatograms of PcBIS assays. The incubation mixtures contained benzoyl-CoA and salicoyl-CoA. Control, heat-denatured protein.
(TIF)

S3 Fig GC-MS analysis of 3,5-dihydroxybiphenyl. The compound was either enzymatically formed (A) or chemically synthesized (B).
(TIF)

S4 Fig GC-MS analysis of 4-hydroxycoumarin. The compound was either enzymatically formed (A) or commercially obtained (B).
(TIF)

S5 Fig Kinetic parameters of PcBIS1. The kinetic properties were determined for benzoyl-CoA and malonyl-CoA (A) and salicoyl-CoA and malonyl-CoA (B).
(TIF)

S6 Fig Kinetic parameters of PcBIS2. The kinetic properties were determined for benzoyl-CoA and malonyl-CoA (A) and salicoyl-CoA and malonyl-CoA (B).
(TIF)
