# Supplementary figures and images for: Expression of Biphenyl Synthase Genes and Formation of Phytoalexin Compounds in Three Fire Blight-Infected Pyrus communis Cultivars
Source: PLoS One. 2016 Jul 13;11(7):e0158713. doi: 10.1371/journal.pone.0158713 (PMC4943715; doi:10.1371/journal.pone.0158713)

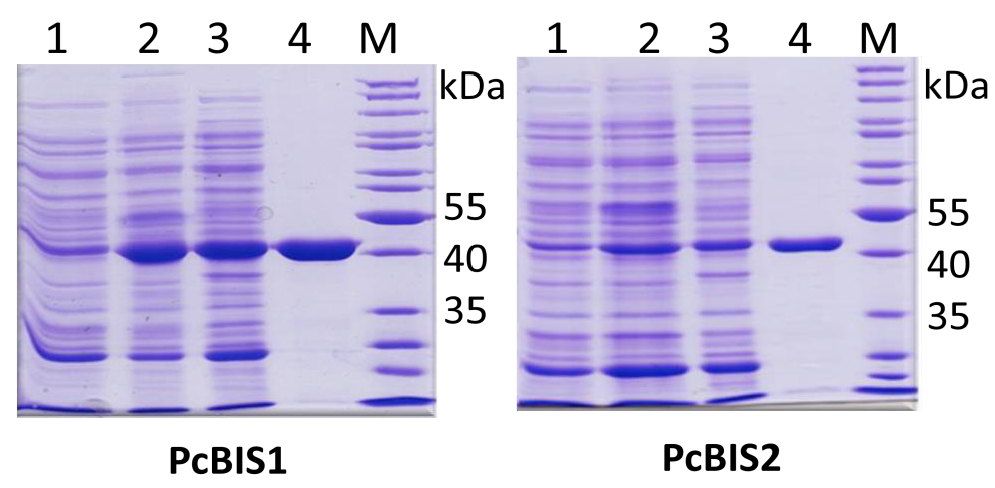

Supplement: S1 Fig — PcBIS1 and PcBIS2 were overexpressed in E. coli and purified by affinity chromatography on Ni-NTA agarose. 1, pre-induction; 2, post-induction; 3, soluble protein; 4, affinity-purified protein; M, protein marker. (TIFF) [file pone.0158713.s001.tiff]

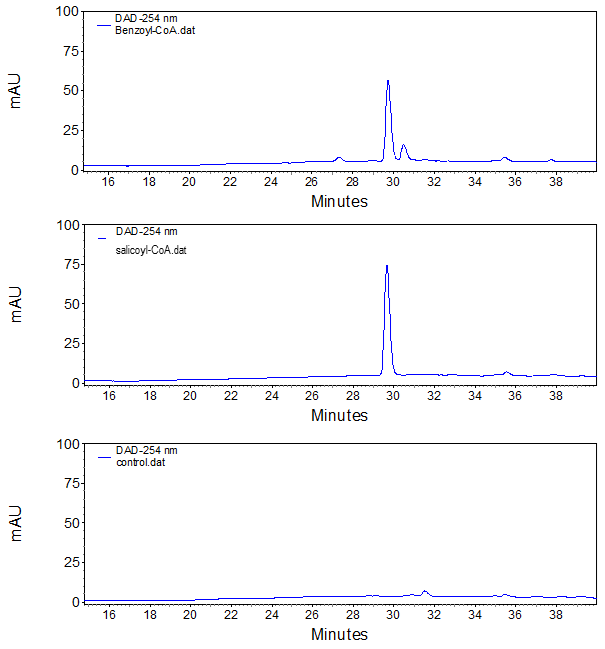

Supplement: S2 Fig — The incubation mixtures contained benzoyl-CoA and salicoyl-CoA. Control, heat-denatured protein. (TIF) [file pone.0158713.s002.tif]

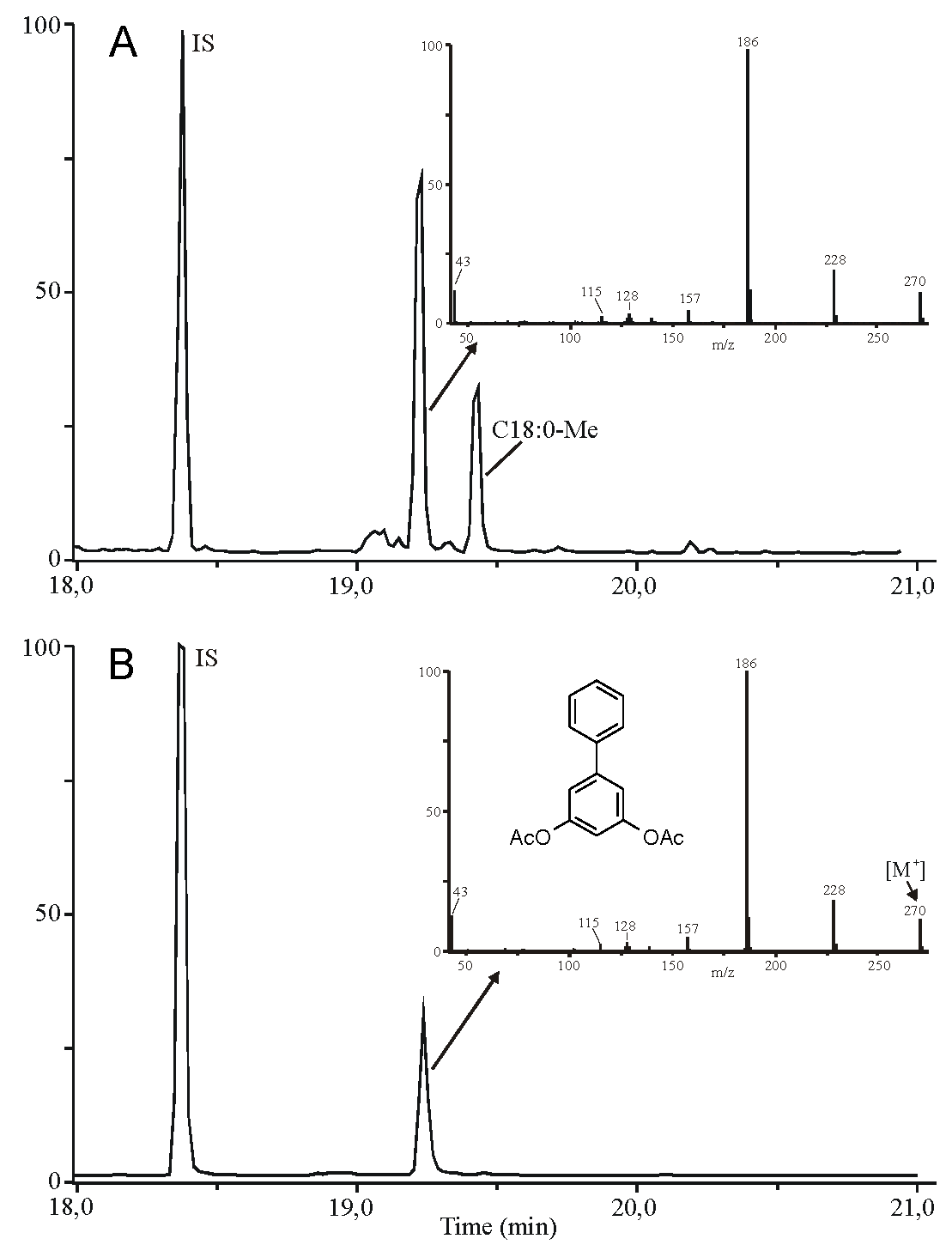

Supplement: S3 Fig — The compound was either enzymatically formed (A) or chemically synthesized (B). (TIF) [file pone.0158713.s003.tif]

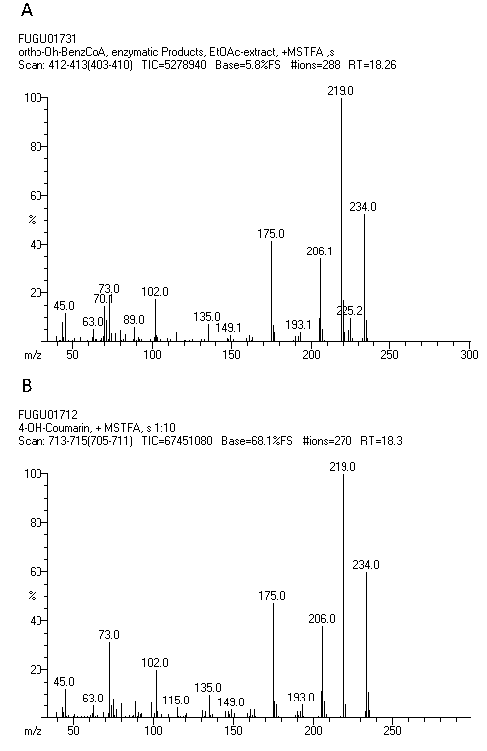

Supplement: S4 Fig — The compound was either enzymatically formed (A) or commercially obtained (B). (TIF) [file pone.0158713.s004.tif]

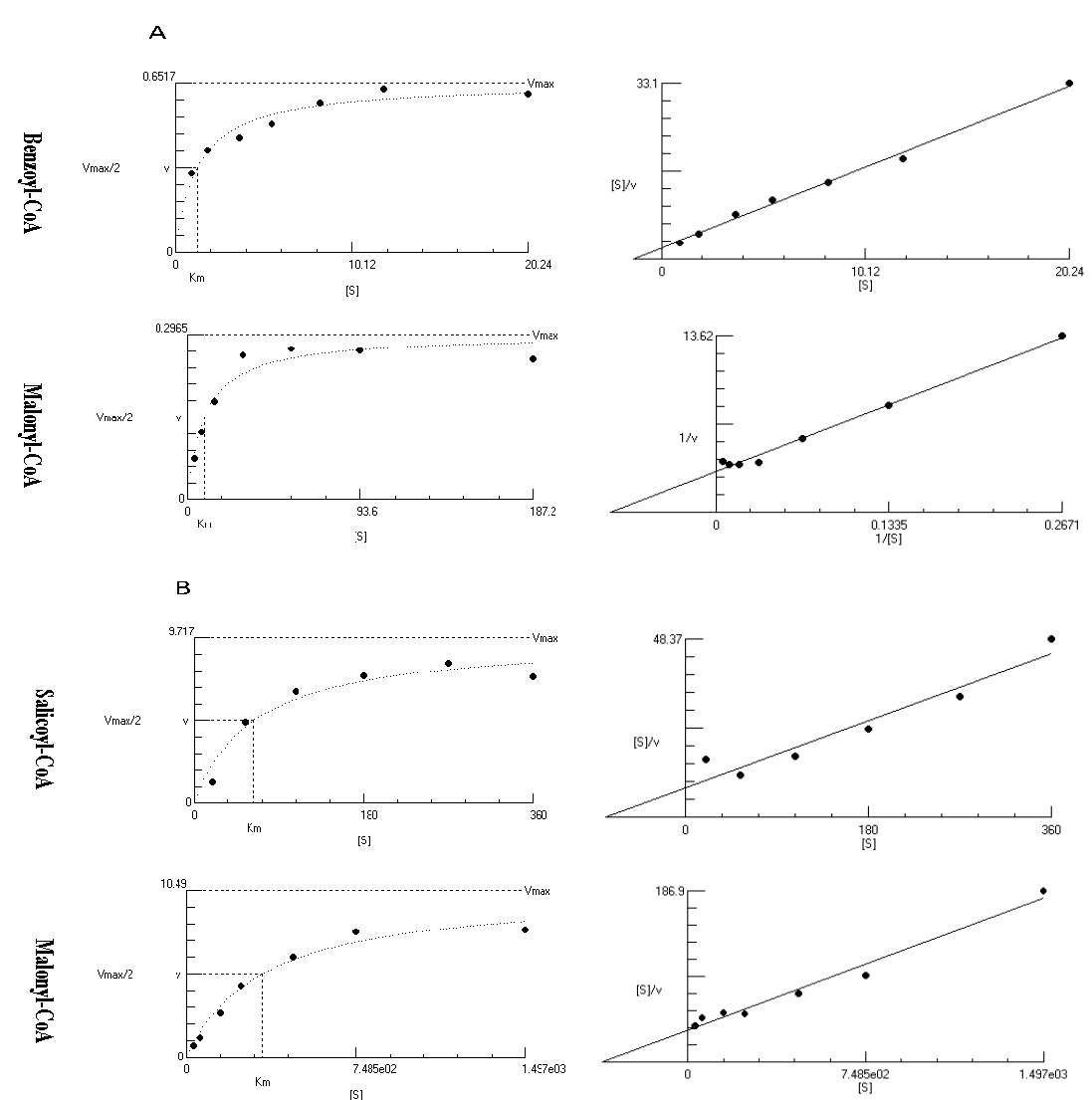

Supplement: S5 Fig — The kinetic properties were determined for benzoyl-CoA and malonyl-CoA (A) and salicoyl-CoA and malonyl-CoA (B). (TIF) [file pone.0158713.s005.tif]

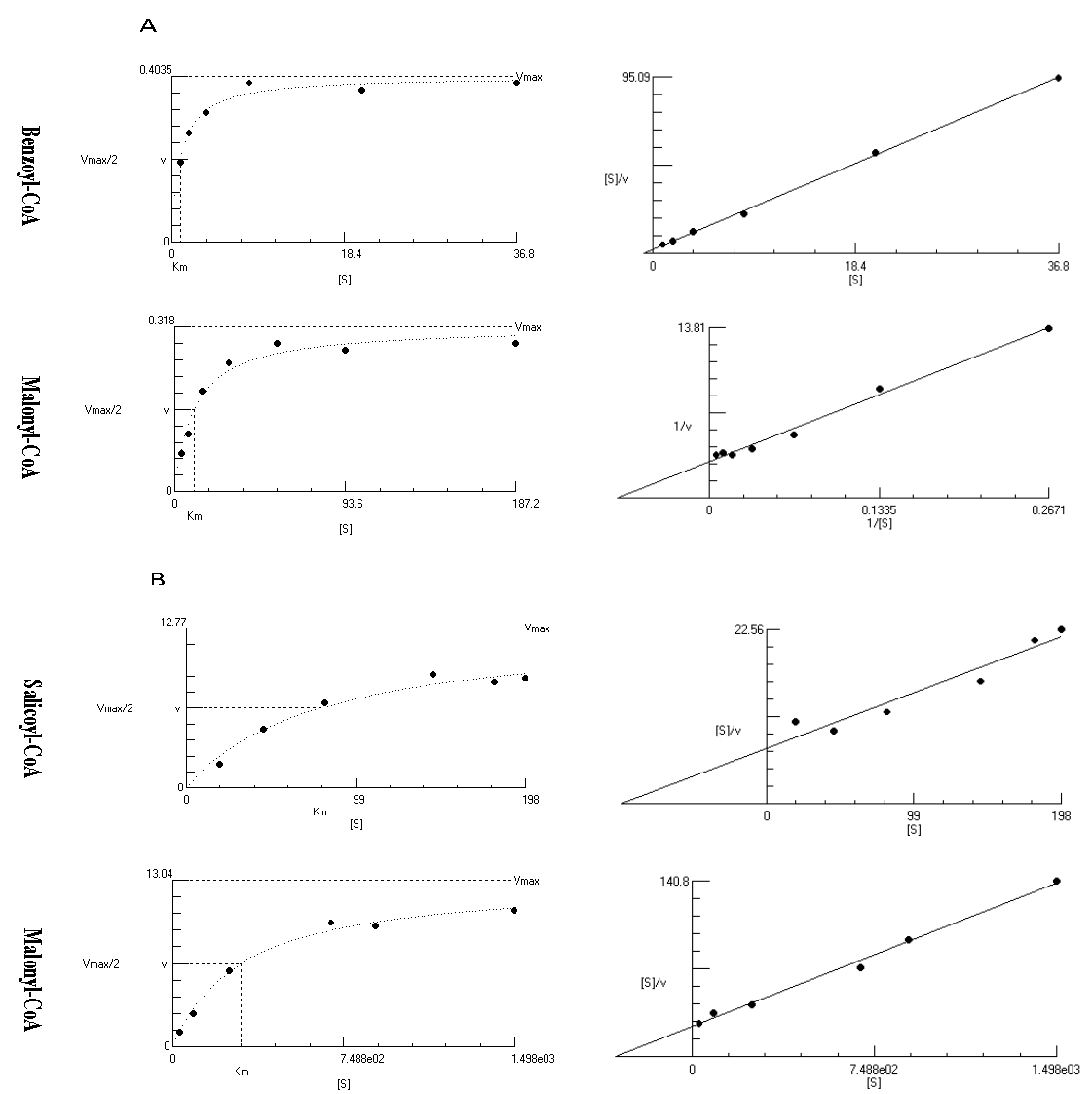

Supplement: S6 Fig — The kinetic properties were determined for benzoyl-CoA and malonyl-CoA (A) and salicoyl-CoA and malonyl-CoA (B). (TIF) [file pone.0158713.s006.tif]
